# Supplementary material for: EnzML: multi-label prediction of enzyme classes using InterPro signatures
Source: BMC Bioinformatics. 2012 Apr 25;13:61. doi: 10.1186/1471-2105-13-61 (PMC3483700; doi:10.1186/1471-2105-13-61)
Supplement: Addtional file 5 — The Java code to format the data files, evaluate and predict. The file enzml_java_code.tar.gz contains the Java code used to format database data to ARFF and XML formats, to execute cross and train-test (jackknife) evaluations and to record evaluation results to database. More information is included in the readme.txt file and the Javadoc files. The code can be used with a MySQL database. To use a different database software, other JDBC drivers might be required. [file 1471-2105-13-61-S5.gz › java_code/enzml2011/doc/uk/ac/ed/inf/csb/taweka/mulan/xml/MulanLabel.html]

MulanLabel


---


|  |  |  |
| --- | --- | --- |
| **Overview** \| **Package** \| **Class** \| **Use** \| **Tree** \| **Deprecated** \| **Index** \| **Help** | |  |
| PREV CLASS   **NEXT CLASS** | **FRAMES**    **NO FRAMES**     **All Classes** |  |
| SUMMARY: NESTED \| FIELD \| CONSTR \| METHOD | DETAIL: FIELD \| CONSTR \| METHOD |  |


---


## uk.ac.ed.inf.csb.taweka.mulan.xml Class MulanLabel

```
java.lang.Object
  uk.ac.ed.inf.csb.utils.webutils.simpledomparser.XmlNode
      uk.ac.ed.inf.csb.taweka.mulan.xml.MulanLabel
```

---

``` public class MulanLabel extends uk.ac.ed.inf.csb.utils.webutils.simpledomparser.XmlNode ```

A node in the Mulan XML (a label for machine learning) \*

```
Version:
:   5 May 2010


Author:
:   Luna De Ferrari luna.deferrari-at-ed.ac.uk


---


Constructor Summary


MulanLabel(java.lang.String labelName)


| Method Summary | |
| --- | --- |
| void | addChildElement(MulanLabel label)              Add a child label to a label |
| void | addChildElement(java.lang.String labelName)              Add a child label to a label |


Methods inherited from class uk.ac.ed.inf.csb.utils.webutils.simpledomparser.XmlNode


addChildNode, getAttributeValue, getChildElements, getTagName, getText, getXmlTreeFromString, getXmlTreeFromUrl, hasChildren, nodeHeadToString, removeAttribute, removeChildNode, setAttribute, setTagName, setText, toString


Methods inherited from class java.lang.Object


equals, getClass, hashCode, notify, notifyAll, wait, wait, wait


Constructor Detail


### MulanLabel


```
public MulanLabel(java.lang.String labelName)
```


Method Detail


### addChildElement


```
public void addChildElement(MulanLabel label)
```


:   Add a child label to a label

    :   Parameters:: label - the child label (with its children etc.)


---


### addChildElement


```
public void addChildElement(java.lang.String labelName)
```


:   Add a child label to a label

    :   Parameters:: labelName - the name of the label to be added


---


|  |  |  |
| --- | --- | --- |
| Overview \| Package \| Class \| Use \| Tree \| Deprecated \| Index \| Help | |  |
| PREV CLASS   NEXT CLASS | FRAMES     NO FRAMES          All Classes |  |
| SUMMARY: NESTED \| FIELD \| CONSTR \| METHOD | DETAIL: FIELD \| CONSTR \| METHOD |  |


---
```
